# Protein phosphatase 2A (PP2A) function in male germ cells and its importance for male fertility

**DOI:** 10.1530/EC-25-0554

**Published:** 2025-10-30

**Authors:** Violaine Simon

**Affiliations:** Institute for Advanced Biosciences, INSERM U 1209, CNRS UMR 5309, Université Grenoble Alpes, Team ‘Physiopathology and Pathophysiology of Sperm Cells’, Grenoble, France

**Keywords:** spermatogenesis, phosphorylation, phosphatase PP2A, male fertility

## Abstract

Infertility is a common issue that affects approximately 30 million men worldwide, most often resulting from defects during spermatogenesis, a complex cellular differentiation process allowing the formation of spermatozoa. During spermatogenesis, a large number of proteins involved in sperm production are phosphorylated, mainly on serine and threonine residues. As recently shown, this phosphorylation process is essential for male fertility. Protein phosphorylation depends on a balance between kinase and phosphatase activity, and although the kinases involved are relatively well characterized, much less is known about the presence and role of serine/threonine phosphatases within male germ cells. The aim of this review is to highlight the importance of protein phosphatase 2A (PP2A) in male fertility through direct expression of several PP2A subunits in male germ cells. First, the review will provide current knowledge on PP2A structure and regulation. Then, the importance of PP2A for spermatogenesis will be illustrated by the description of gene mutations and deletions recently identified in PP2A that result in male infertility, both in humans and mice. The review will also provide information on the level and stage of expression of PP2A subunits and endogenous inhibitors in human male germ cells, indicative of their dynamic regulation throughout spermatogenesis. Finally, the review will explore the involvement of PP2A beyond spermatogenesis during sperm maturation processes. Overall, this review highlights the critical functions of PP2A in male germ cells, reinforcing the importance of investigating the potential pathogenic deregulation of PP2A activity in cases of human male infertility.

## Introduction

Infertility is a common issue that affects approximately 15% of couples worldwide, with 20–30% of cases resulting directly from male factors. Hence, it is estimated that at least 30 million men of reproductive age suffer from infertility worldwide (1). In this context, a better understanding of the process of spermatogenesis and the associated mechanisms of regulation is requisite for the potential development of novel therapeutics for male infertility.

During spermatogenesis, the formation of structurally formed spermatozoa occurs within the seminiferous tubules of the testis following a complex cell differentiation process (for review see (2)). The initial phase of spermatogenesis consists of spermatocytogenesis, during which spermatogonial stem cells (SSCs) differentiate into proliferating spermatogonia that then enter meiosis as spermatocytes, which then undergo two meiotic divisions to generate haploid spermatids. SSCs undergo successive mitotic divisions resulting in either self-renewal or differentiation. Once differentiated, spermatogonia enter the prophase of the first meiotic division to become spermatocytes I. Spermatocytes I achieve their first meiotic division to generate two haploid spermatocytes II, which progress to meiosis II to give rise to four round haploid spermatids. During spermiogenesis, the last step of spermatogenesis, the round spermatids undergo morphological changes in which the cytoplasm is reduced, the DNA is compacted, and the specialized structures required for fertilization, such as the flagellum and the acrosome, are formed. At the end of the process, the spermatozoa are released into the lumen of the tubule and acquire the capacity to fertilize the oocyte following functional maturation during their journey through the male and female genital tracts (for review see (3)). As a result, defects in any of the steps described above can preclude proper fertilization and represent the most common factors of male infertility.

Spermatogenesis is a finely regulated process involving hormonal regulation through the hypothalamic–pituitary–testicular axis, which is well documented (4). In addition, other regulatory processes, such as phosphoregulation, are involved. The importance of sperm protein phosphorylation in fertility is highlighted by recent observations of its alteration in spermatozoa from infertile men (for review see (5)). Hence, phosphoregulation is highly active during spermatogenesis, as reported in large-scale human and mouse phosphoproteomic studies (6, 7, 8). Phosphoproteins are highly abundant, representing up to one-third of the testis proteome (6), and many are closely related to sperm production and development (6, 7, 8). Phosphorylation mainly occurs on serine (∼85%) and threonine (∼10%) residues and is driven by several serine/threonine (Ser/Thr) kinases that are highly active during spermatogenesis (6, 7, 8). These kinases include cyclin-dependent kinases (CDKs) and p21-activated kinases (PAKs), as well as mitogen-activated protein kinases (MAPKs), polo-like kinases (PLKs), and testis-specific serine kinases (TSSKs), for which a role in spermatogenesis has been demonstrated (for review see (9)). The state of protein phosphorylation not only results from phosphorylation by these kinases but also from the converse enzymatic action of phosphatases, which remove phosphate anions. Unlike Ser/Thr kinases, the presence and role of Ser/Thr phosphatases have been much less addressed in male germ cells. Ser/Thr phosphatases are composed of three different families: phosphoprotein phosphatases (PPP), protein phosphatase metal-dependent (PPM), and FCP/SCP aspartate-dependent phosphatases. PPPs are the best characterized and most abundant phosphatases in cells; they include protein phosphatases 1 (PP1), 2A (PP2A), and 2B (PP2B) and protein phosphatases 4, 5, 6, and 7 (PP4, PP5, PP6, PP7) (10). PP2A, together with PP1, is responsible for most Ser/Thr dephosphorylation in most cell types. PP1 is essential for male fertility, notably through the regulation of spermiogenesis (11, 12). While the role of PP1 in male fertility has been the focus of numerous studies (for reviews see (13, 14, 15)), data regarding the involvement of PP2A in male germ cell production remain sparse and fragmented. This review aims to synthesize current knowledge on PP2A to highlight its functions throughout the different stages of spermatogenesis and beyond in the course of sperm functional maturation, overall stressing its essential roles in male fertility.

This review will: i) summarize current knowledge of PP2A and its role in mitosis and meiosis, two processes essential for the production of male germ cells; ii) shed light on the impact of the deregulation of PP2A function on spermatogenesis and male fertility; iii) provide, for the first time, exhaustive characterization of the expression of PP2A and its endogenous inhibitors in human male germ cells during spermatogenesis; and iv) explore the role of PP2A beyond spermatogenesis in the course of sperm maturation.

## Phosphatase PP2A: structure and regulation

### Structure and biogenesis of PP2A

**Figure 1:**
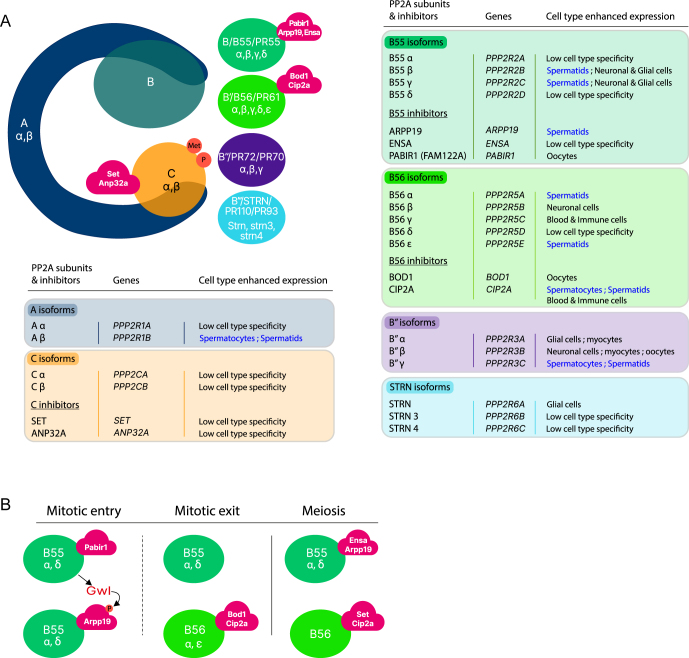
PP2A heterotrimers: structure, expression, and involvement in mitosis and meiosis. (A) The PP2A core enzyme is composed of the A and C subunits. This dimer binds to distinct B-regulatory subunits from the four following families: B55, B56, PR, and STRN. Certain subunits (C, B55, and B56) can interact with specific endogenous inhibitors: SET and ANP32A (C-subunit), PABIR1, ARPP19, and ENSA (B55), and BOD1 and CIP2A (B56). The association of specific B subunits with the core enzyme is regulated by methylation (Met) and phosphorylation (P) of the C-terminal portion of the C-subunit. Each PP2A subunit family contains several isoforms that show either low cell-type specificity or enhanced expression in a given cell type of the human body (https://www.proteinatlas.org/). Certain PP2A inhibitors also show enhanced expression in specific cell types. (B) Involvement of the PP2A-B55 and PP2A-B56 subunits, together with their endogenous inhibitors, in mitosis and meiosis (for reviews see (27, 45)). Entry into mitosis is triggered by the initial inhibition of PP2A-B55 by PABIR1, leading to the activation of Gwl kinase and the phosphorylation of ARPP19, which then binds to and inhibits PP2A-B55 to maintain high levels of protein phosphorylation (29). At the metaphase-to-anaphase transition, there is a decrease in Gwl kinase activity that leads to the reactivation of PP2A-B55, which is essential for the dephosphorylation of the mitotic substrates required for the exit from mitosis. PP2A-B55, together with PP2A-B56, BOD1, and CIP2A, promotes the exit of cells from mitosis. Both the PP2A-B55 and PP2A-B56 phosphatases, together with their endogenous inhibitors, promote the progression of oocytes into meiosis (for review see (45)). Among the various PP2A-B55 isoforms, isoforms α and δ play an essential role in the timely entry into and exit from mitosis (69) and in the progression of oocytes into meiosis I (70, 71).

PP2A is a heterotrimer composed of three different subunits: a scaffold subunit (A), a variable regulatory subunit (B), and a catalytic subunit (C) (Fig. 1A). The scaffold subunit is formed by 15 tandem repeats of the Huntington-elongation-A subunit-TOR (HEAT), each composed of a pair of antiparallel α helices, conferring a horseshoe-shaped structure. The C-terminal portion of the A subunit (HEAT repeats 11–15) associates with the C subunit to form the core enzyme. There are two isoforms, α and β, for the scaffold and catalytic subunits, which share strong sequence similarity (10). Most of the core dimers in cells associate with a structurally distinct regulatory B subunit, which determines the substrate specificity, regulation, and subcellular localization of the resulting heterotrimeric PP2A complex. There are fifteen human genes that encode the B subunits, classified into four families, with very little sequence similarity and no structural similarity between families (10): B/B55/PR55, B′/B56/PR61, B″/PR72/PR70, and B‴/STRN/PR110/PR93 (Fig. 1A). Distinct isoforms have been identified for each family: B55 (α, β, γ, δ), B56 (α, β, γ, δ, ε), B″/PR (α, β, γ), and striatin (STRN, STRN3, STRN4), and some have splice variants. Thus, the large repertoire of regulatory B subunits gives rise to at least 80 different PP2A heterotrimers with specific physiological functions. The PP2A-B55 subunit, composed of WD40 repeats and forming a seven-bladed β propeller, interacts with HEAT 1–7 of the A subunit, whereas the PP2A-B56 subunit, containing eight HEAT-like repeats, interacts with HEAT 2–8 of the A subunit (16). In addition, the B56 subunit interacts strongly with the C subunit of PP2A, whereas the B55 subunit interacts more loosely (16). The B″ subunit, composed of Ca2^+^-binding EF motifs, and the atypical B‴ subunit, belonging to the multicomplex STRIPAK (striatin-interacting phosphatase and kinase), also establish several contacts with the N-terminal HEAT domains of the A subunit (17). The association of specific B subunits with the dimeric core enzyme is regulated in cells by methylation and phosphorylation of the C-terminal portion of the PP2A catalytic subunit. Methylation of leucine 309, catalyzed by leucine carboxyl methyltransferase-1 (LCMT-1), promotes the binding of methylation-sensitive B subunits, such as B55α and B56 α, β, and ε, whereas phosphorylation of threonine 304 prevents the formation of the PP2A-B55 holoenzyme and decreases binding to the PP2A-B56 α, β, and ε subunits (for reviews see (18, 19)).

### PP2A substrate specificity

One way for PP2A heterotrimers to achieve substrate specificity is through the recognition of substrates via specific docking motifs in B subunits. These motifs have only been identified for the two largest B families, B55 and B56, and the mechanisms that dictate substrate specificity for the B″ and B‴ families are still unknown. A conserved hydrophobic binding pocket present on all isoforms of PP2A‐B56 regulatory subunits binds to a short linear interaction motif (SLiM), denoted as LxxIxE (where x represents any amino acid), present in intrinsically disordered regions of proteins (20, 21). In addition, another conserved acidic domain in B56 subunits mediates interactions with positively charged motifs in close proximity to the LxxIxE motif (22). Recent studies suggest that the isoforms of PP2A-B56, which differ only by their N- and C-termini, show distinct substrate specificity. The sequence EPVA, present within the C-termini of B56α and B56ε but absent from other B56 isoforms, mediates B56α-specific interactions with shugoshin 2 (23). In contrast to PP2A-B56, the mechanism of binding of PP2A-B55 with specific substrates does not involve SLiMs, but rather key amino acids in the α helices of substrates that engage discrete hydrophobic and electrostatic patches within PP2A-B55 (24, 25). In addition to distinct substrate specificity, PP2A-B55 and PP2A-B56 dephosphorylate distinct sites in substrates (26). PP2A-B55 efficiently dephosphorylates serine–proline and threonine–proline, whereas PP2A-B56 works only poorly on proline-directed phosphorylation sites (26). Thus, the type of B subunit and isoform in PP2A heterotrimers determines PP2A activity in the cell (26).

### Endogenous inhibitors of PP2A

The activity of PP2A-B55 and PP2A-B56 heterotrimers can be inhibited in cells by the selective binding of endogenous inhibitors that differentially associate with each B subunit (Fig. 1A).

ARPP19 (cAMP-regulated phosphoprotein 19) and ENSA (alpha-endosulfine) are small, disordered, heat-stable proteins that specifically bind and inhibit PP2A holoenzymes containing the B55 regulatory subunit (for review see (27)). They are phosphorylated by the mitotic kinase Greatwall (Gwl), which transforms them into potent inhibitors of PP2A-B55. PABIR1 (PP2A-Aα and B55α interacting regulator 1), also called FAM122A, was recently identified as an interactor and inhibitor of the PP2A-B55 complex (28). In contrast to ARPP19 and ENSA, PABIR1 inhibits PP2A-B55 in a phosphorylation-independent manner (28). Cryoelectron microscopy and structural modeling of the B55 inhibitors ARPP19 and FAM122A revealed that, similar to the B55 substrates (25), these inhibitors contain α helices that mediate binding to B55 (29, 30, 31).

The proteins BOD1 (biorientation defective 1) and CIP2A (cancerous inhibitor of PP2A) are specific inhibitors of PP2A-B56. BOD1, a disordered heat-stable protein, was first identified as an inhibitor of the kinetochore-associated PP2A-B56 and is required for chromosome biorientation at mitosis (32, 33). Later, a novel role of BOD1 was discovered in postmitotic neurons, in which it contributes to the development and maintenance of cognitive features (34). CIP2A was first discovered as an oncogenic inhibitor of PP2A and is overexpressed in many human cancers (for review see (35)). Upon direct binding of CIP2A to the PP2A-B56 heterotrimer, CIP2A displaces the PP2A-A subunit and shields the LxxIxE-motif substrate-binding pocket on B56, preventing substrate dephosphorylation (36).

To date, no endogenous inhibitors for the B″ and B‴ families have been identified.

The catalytic subunit of PP2A is directly inhibited through interaction with the proteins ANP32A (acidic nuclear phosphoprotein 32) and SET (37, 38, 39). ANP32A, also called inhibitor 1 of PP2A, inhibits PP2A-dependent dephosphorylation of the tau protein in the brain (38). Its expression is upregulated in the brains of patients with Alzheimer’s disease (40) and is responsible for the abnormal hyperphosphorylation of tau (41). SET, also called inhibitor 2 of PP2A was first identified in a patient with acute undifferentiated leukemia resulting from translocation of the *Set* gene into the *Can* gene (42). Phosphorylation of Set alters its activity (43) and its ability to inhibit PP2A (for review see (44)). The deregulation of SET is associated with increased inhibition of PP2A activity in many types of cancer and in Alzheimer’s disease (for review see (44)).

## PP2A function during spermatogenesis – importance for male fertility

It is well established that PP2A plays crucial roles in regulating the cell cycle in somatic cells and during female gametogenesis. In particular, the contribution of PP2A-B55 and PP2A-B56, together with their endogenous inhibitors, was well documented in these processes (for reviews see (27, 45); Fig. 1B). The successive inhibition of PP2A-B55 by PABIR1 and ARPP19 is required for the entry of cells into mitosis (Fig. 1B). At the metaphase-to-anaphase transition, PP2A-B55 is reactivated and, together with PP2A-B56, BOD1, and CIP2A, promotes the exit of cells from mitosis. At meiosis, both the PP2A-B55 and PP2A-B56 phosphatases, together with their endogenous inhibitors, regulate spindle formation and chromosome segregation in oocytes (Fig. 1B).

**Figure 2:**
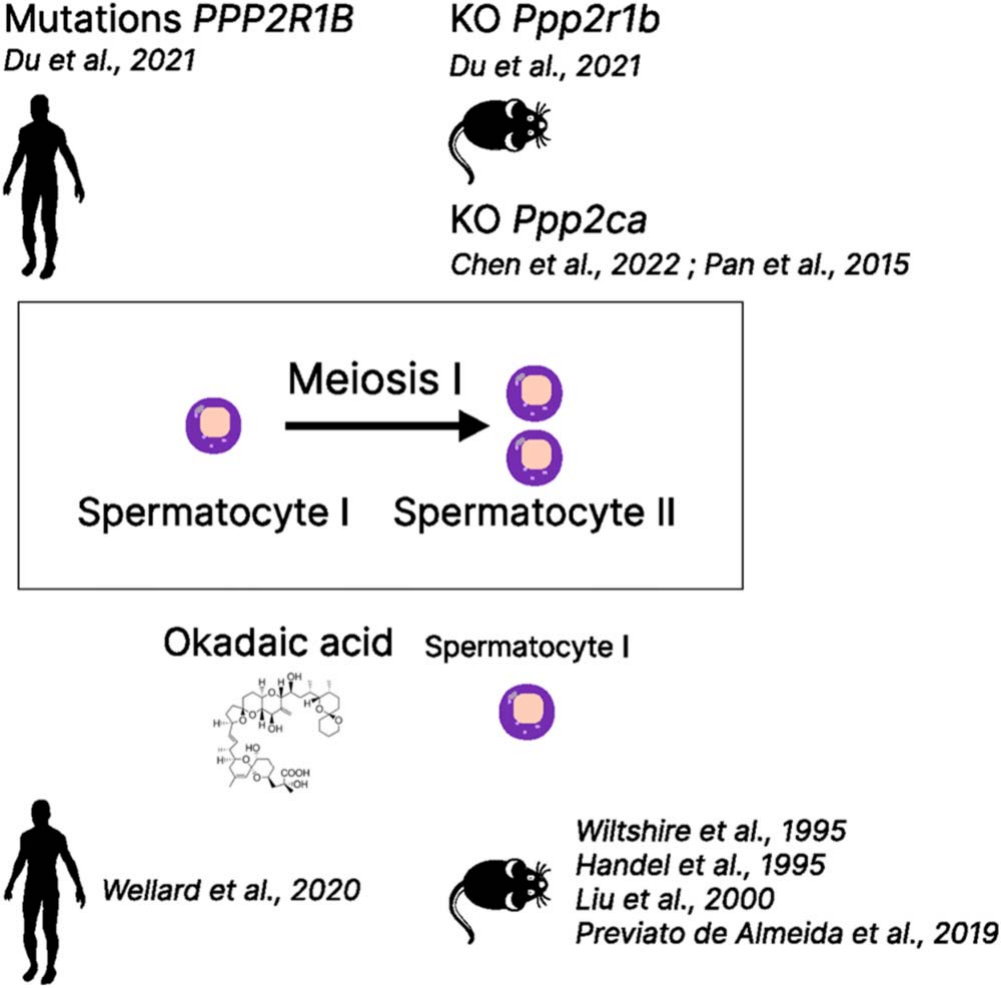
PP2A is required for the progression of meiosis I of human and mouse male germ cells. Disruption of PP2A activity by inhibition with okadaic acid, gene mutation, or gene deletion leads to meiotic arrest of human and mouse spermatocytes I and subsequent azoospermia and male infertility.

In contrast, the role of PP2A for proper sperm production, functionality, and overall male fertility remains underappreciated. In the following chapter, several studies are described to highlight a critical role of PP2A for spermatogenesis and male fertility through analysis of the impact of PP2A pharmacological inhibitors and PP2A subunit gene mutations/deletions in humans and mice (Fig. 2).

### Impact of pharmacological PP2A inhibitors on spermatogenesis

A role for Ser/Thr phosphatases in male reproduction is suggested by studies that evaluated the impact of microcystin toxins (MCs) on reproductive function (for review see (46)). MCs are produced by freshwater cyanobacteria and exert their toxicity primarily through the inhibition of phosphatases PP1 and PP2A. Humans are mainly exposed to MCs through oral or dermal contact with water subject to a toxic bloom of cyanobacteria (for review see (47)). MCs can be found in the serum of chronically exposed human populations (48, 49) and cause damage to organs such as the liver (for review see (47)). MCs are also found in the semen of men chronically exposed to MCs (50), and their levels in semen correlate with decreased sperm counts and increased sperm head abnormalities (50). The impact of MCs on spermatogenesis has also been observed in other mammals, as well as in amphibians, fish, and worms (for review see (46)). In rodents, MCs preferentially accumulate in the liver and testis, where they localize in germ cells and Sertoli cells (51) and affect sperm quality similarly to human exposure (decreased sperm concentration and increased sperm abnormality rate) (for review see (46)).

Pioneering work from Handel’s group showed that the proper course of meiosis in primary spermatocytes is directly driven by Ser/Thr phosphatase expressed in male germ cells. Indeed, inhibition in mouse primary spermatocytes of the phosphatases PP1 and PP2A with a high concentration of okadaic acid (OA, 5 μM; OA: PP2A, IC_50_ = 0.1 nM, PP1, IC_50_ = 15–20 nM) induces a premature entry of pachytene spermatocytes into metaphase I (Handel *et al.* (52); Wiltshire *et al.* (53)). This was observed not only in mouse spermatocytes (52, 53, 54, 55) but also in rat (56) and human spermatocytes (57). To date, only one study has reported that the selective inhibition of PP2A with a very low concentration of OA (0.7 nM) disrupted the development of male germ cells in the lily plant, resulting in the sterility of lily pollen grains (58). In mammals, however, the concentrations of phosphatase inhibitors used have not allowed determination of the specific contribution of PP2A to male germ cell development. Transgenic mice in which the PP2A and PP1 genes are not expressed in male germ cells are the only models currently providing information on the respective contributions of these phosphatases to germ cell development during spermatogenesis.

### Impact of PP2A gene deletion and mutation on mouse and human spermatogenesis

Targeted deletion in testicular germ cells of the gene encoding the catalytic subunit of PP2A (isoform α, *PPP2CA*) or the gene encoding the catalytic subunit of PP1 (isoform γ, *PPP1CC*) leads to spermatogenesis defects and male infertility in both cases (11, 59, 60). However, spermatogenesis defects are not the same depending on the nature of the targeted phosphatase. *Ppp2ca*^−/−^ mutant mice exhibit azoospermia, which results from an early arrest of spermatogenesis at the pachytene stage of prophase of the first meiotic division (59, 60). *Ppp1cc*^−/−^ mutant mice, on the other hand, exhibit oligo-teratozoospermia (11). Unlike PPP2CA, absence of PPP1CC did not inhibit the progression and completion of meiosis in male germ cells, as demonstrated by the presence of spermatids in the seminiferous tubules of mutant mice. However, some polyploid and aneuploid spermatids were observed in *Ppp1cc*-deficient mice, indicating chromosome segregation defects during meiosis of a small number of germ cells (12, 61). The primary disruption of spermatogenesis in *Ppp1cc*^−/−^ mutant mice likely occurs during spermiogenesis, as shown by a deficit of elongating and condensing spermatids in the seminiferous tubules of *Ppp1cc*^−/−^ mutant mice (61) and an increase in the percentage of morphologically abnormal spermatozoa in the caudal epididymis of *Ppp1cc*^−/−^ mutant mice (11). Altogether, these two studies indicate that PP2A has an essential role in the progression and completion of male germ cell meiosis, while PP1 plays a role in spermatid differentiation.

The importance of PP2A in male germ cells for meiosis is further supported by the phenotype of mice with homozygous deletion of the gene coding the isoform β of the PP2A scaffolding subunit (*Ppp2r1b*). Deletion of this gene led to male infertility with a phenotype of azoospermia (62). Interestingly, the testes from *Ppp2r1b*^−/−^ mutant mice contained an accumulation of spermatocytes arrested at the pachytene stage of meiosis I, which likely results from impairment of the interhomolog crossover required for proper segregation of homolog chromosomes ((62); Fig. 2). In other non-mammalian organisms, such as plants and insects, deletions of PP2A regulatory B subunits (B and B′) cause severe male sterility with a defect in sister chromatid segregation during meiosis in *Arabidopsis thaliana* (63, 64) and in *Drosophila* (65).

**Figure 3:**
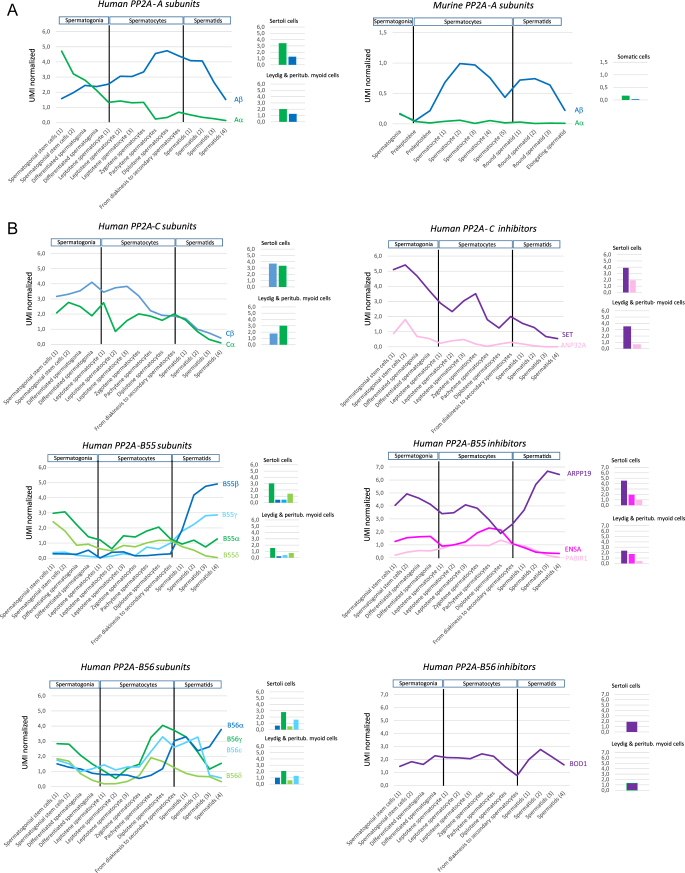

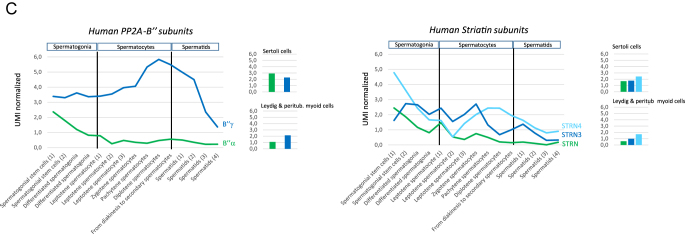
Transcriptomic data for PP2A subunits and their endogenous inhibitors in the testes. Analysis of single-cell RNA quantitative sequencing datasets from human (A, B, C) and mouse (A) adult testis, available in Wang *et al.* (67) and Green *et al.* (66), respectively. Transcript levels of endogenous PP2A inhibitors and isoforms of the PP2A scaffold, catalytic, and regulatory subunits (B55, B56, B″, and striatin) are indicated by UMI (unique molecular identifier) counts, with each UMI representing one transcript. Expression levels were normalized by dividing each gene’s UMI count by the total UMI count in the corresponding cell for all stages of germ cell differentiation (from spermatogonia to differentiated spermatids) and for somatic cells (Sertoli, Leydig and peritubular myoid cells).

In humans, Du and colleagues recently reported heterozygous missense mutations in the *PPP2R1B* gene in three unrelated infertile Chinese men with male germ cells in meiotic arrest ((62); Fig. 2). One of these infertile patients, diagnosed with non-obstructive azoospermia (NOA), originated from a family with four generations exhibiting a dominant transmission mode of infertility. Whole-exome sequencing of blood samples obtained from six members of the family, including the infertile patient, identified the heterozygous missense mutation c581G (p.R194Q) within the *PPP2R1B* gene. This mutation, with a low allelic frequency in the general population (0.000004), co-segregates with male infertility and is absent from patients with a normal sperm concentration (62). As demonstrated in biochemical studies, the c581G (p.R194Q) mutation induces degradation of both wild-type and mutant PPP1R1B proteins and, accordingly, PPP2R1B is nearly absent from the seminiferous tubules of the patient (62). As observed for the *Ppp2r1b*^−/−^ mutant mice, the testes from the patient contain an accumulation of primary spermatocytes and no spermatids (62). Overall, PPP2R1B expression appears to be critical for the success of human and mouse spermatogenesis, notably by enabling completion of the first meiotic division of male germ cells. Analysis of *PPP2R1B* transcript levels in single-cell quantitative RNA-seq data from germ cells at different stages of spermatogenesis indicates a strong increase in *PPP2R1B* gene expression during prophase of the first meiosis in human and mouse spermatocytes (Fig. 3A, PP2A-Aβ; (66, 67)). Of note, spermatocytes are the cells that express the highest level of the *PPP2R1B* transcript in the human body (https://www.proteinatlas.org/ENSG00000137713-PPP2R1B; Fig. 1). The upregulation of *PPP2R1B* expression in spermatocytes may therefore be a key event required for the progression of spermatocytes into meiosis I.

## Expression of PP2A subunits and inhibitors during spermatogenesis

To date, no study has characterized the expression of the different PP2A subunits in the testis. Based on the single-cell quantitative RNA-seq data from germ cells at different stages of spermatogenesis (66, 67), the next chapter of this review provides the first characterization of the PP2A subunit transcripts expressed in the human testis and also shows that the expression of PP2A subunits and their endogenous inhibitors is dynamically regulated in male germ cells throughout spermatogenesis.

### PP2A scaffold (A) subunits

Unlike the *PPP2R1B* transcript, the *PPP2R1A* transcript encoding isoform α of the PP2A scaffolding subunit is weakly or not expressed in human and murine spermatocytes and spermatids (Fig. 3A, PP2A-Aα). By contrast, the *PPP2R1A* transcript is expressed in spermatogonia and testicular somatic cells, indeed to a greater extent than the *PPP2R1B* transcript (Fig. 3A). This suggests that PP2A heterotrimers are preferentially formed in spermatocytes and spermatids by the PP2A-Aβ subunit and in spermatogonia by the two isoforms of the A-subunit. In contrast to *PPP2R1B,* which has enriched expression in spermatocytes and spermatids, *PPP2R1A* is similarly expressed in all cells of the human body (https://www.proteinatlas.org/ENSG00000105568-PPP2R1A). As demonstrated in the mouse *ppp2r1a*^−/−^ knockout, PP2R1A plays an essential role in embryonic development, the regulation of adult tissue function, oocyte meiosis, and female fertility (for review see (68)). In contrast to the pleiotropic roles of PP2R1A, the role of the PPP2R1B subunit appears to be limited to the testis, as deletion of the gene does not visibly affect the proper development of mice into adulthood or female fertility (62).

### PP2A catalytic (C) subunits and inhibitors SET/ANP32A

In contrast to the specific expression pattern of the isoforms that encode the scaffold subunits, expression of the α and β isoforms of the PP2A catalytic subunit is quite similar in all cells of the human body (https://www.proteinatlas.org/ENSG00000113575-PPP2CA and https://www.proteinatlas.org/ENSG00000104695-PPP2CB) and in testicular cells (Fig. 3B). Somatic and germ cells of the human testis also express transcripts of the two endogenous inhibitors of the PP2A-C subunit, i.e., SET and ANP32A, with greater expression of the SET transcript in humans (Fig. 3B) and mice (not illustrated; (66)). In contrast to SET, which is expressed from the spermatogonia to spermatid stage, the ANP32A inhibitor is solely expressed in spermatogonia.

### PP2A regulatory (B) subunits and their inhibitors

Similar to the PP2A isoform Aβ, five isoforms of the PP2A-B subunits show remarkably enhanced expression in human male germ cells (Fig. 1A): isoforms β and γ of the B55 regulatory subunit (B55β and B55γ), isoforms α and ε of the B56 regulatory subunit (B556α and B56ε), and isoform γ of the B″ regulatory subunit (B″γ). These isoforms are more highly expressed in spermatids, as well as in spermatocytes for the B″γ isoform. Their potential function in germ cells awaits further investigation.

#### PP2A-B55 and ARPP19/ENSA/PABIR1

The various PP2A-B55 isoforms are all expressed in the human testis, but with a distinct expression profile that allows their classification into two groups. The B55α and B55δ isoforms are highly expressed in testicular somatic cells, as well as in spermatogonia and spermatocytes, whereas the spermatid-enriched isoforms, B55β and B55γ, are only expressed in spermatids (Fig. 3B). A similar expression profile of PP2A-B55 isoforms is also found in mice, except for B55γ, which is not expressed in mouse testicular cells (not illustrated; (66)). The three endogenous inhibitors of PP2A-B55, ARPP19, ENSA, and PABIR1, are all expressed in human testicular somatic and germ cells. During spermiogenesis, expression of the *ARPP19* gene increases markedly to reach a high level in spermatids (Fig. 3B), which corresponds to the highest level of Arpp19 expression in the human body (https://www.proteinatlas.org/ENSG00000128989-ARPP19; Fig. 1A). By contrast, gene expression of ENSA and PABIR1 markedly decreases during spermiogenesis to reach undetectable levels by the end. Altogether, this suggests that, within the B55 family, the B55α and B55δ isoforms, together with ARPP19, ENSA, and PABIR1, may play a preferential role during the mitosis and meiosis of male germ cells, as previously shown in other cell types ((69, 70, 71); Fig. 1B). The B55β and B55γ isoforms, together with ARPP19, may have a preferential role during spermiogenesis. This is supported by the results of a recent study that identified PP2A-B55β as a driver gene for spermatid maturation in bulls and boars (72).

#### PP2A-B56 and BOD1/CIP2A

All PP2A-B56 isoforms, except isoform β, are expressed in human and murine testicular somatic and germ cells (Fig. 3B; not illustrated from (66)). Expression of PP2A-B56 isoform transcripts is upregulated in human male germ cells during the first meiotic division and then progressively decreases during spermiogenesis, except for the B56α isoform (Fig. 3B). Instead, its expression increases during spermiogenesis, together with the expression of BOD1, suggesting that the B56α/BOD1 regulatory protein complex may play a role in spermiogenesis. Surprisingly, CIP2A, the other endogenous inhibitor of PP2A-B56, was not detected in the single-cell RNA-seq dataset from Wang *et al.* (67). However, its transcript is detected in other human single-cell RNA-seq data and its expression increases during mitosis and meiosis (https://www.proteinatlas.org/ENSG00000163507-CIP2A); number of transcripts per million: spermatogonia (19), spermatocytes (39), early spermatids (109), late spermatids (9)). This suggests that CIP2A may have a preferential role in regulating PP2A-B56 activity during the mitosis and meiosis of human male germ cells. This is supported by the altered proliferation of spermatogonial progenitors and sperm production in male mice depleted of CIP2A (73). Thus, CIP2A-dependent inhibition of PP2A is likely required for the mitosis of spermatogonia.

#### PP2A-B″

Two isoforms of PP2A-B″, isoforms α and γ, are expressed in human (Fig. 3C) and murine (66) testicular somatic and germ cells. In both species, the PP2A-B″ α and γ isoforms are expressed in spermatogonia, but only the γ isoform is expressed in spermatocytes and spermatids. Expression of PP2A-B″ γ increases significantly in human and murine male germ cells during meiosis and remains high during spermiogenesis (Fig. 3C; not illustrated (66)). Homozygous variants in the human *PPP2R3C* gene lead to the disruption of testis development in syndromic girls with 46,XY complete gonadal dysgenesis (74). Interestingly, the fathers of the affected girls, who were heterozygous for the deleterious PPP2R3C variants, showed severe sperm head, acrosomal, and nuclear abnormalities (74), suggesting that the poorly characterized PP2A-B″ γ subunit is essential for spermatid maturation.

#### PP2A-STRN

All three isoforms of PP2A-STRN (STRN, STRN3, and STRN4) are expressed in somatic and germ cells of the human and murine testes (Fig. 3C; not illustrated (66)). In both species, all three isoforms are expressed in spermatogonia, but only the expression of the STRN3 and STRN4 isoforms increases in germ cells during meiosis. By contrast, the STRN isoform in both humans and mice is only very weakly expressed in spermatocytes and spermatids.

**Figure 4:**
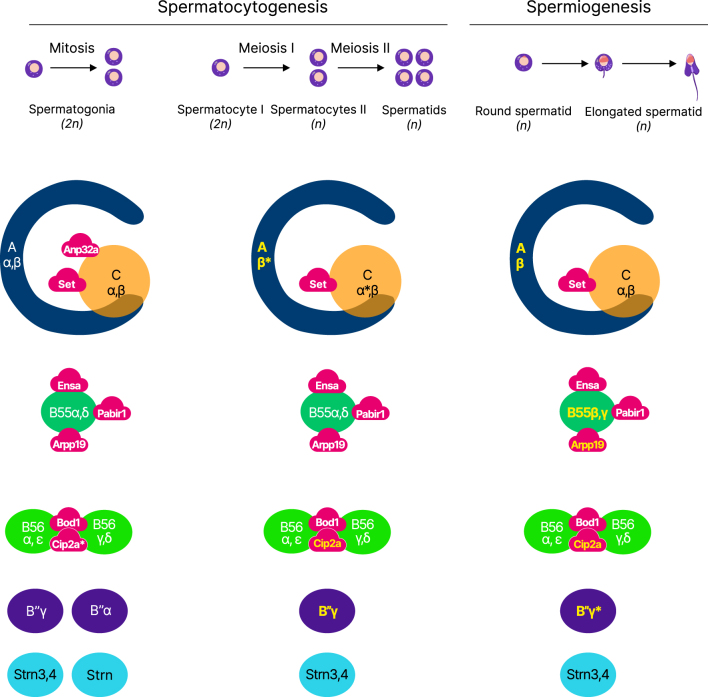
Characterization of PP2A subunits and their inhibitors in human male germ cells undergoing mitosis, meiosis, and spermiogenesis. Representation of PP2A subunits and endogenous inhibitors throughout spermatogenesis showing their expression levels during mitosis, meiosis, and spermatogenesis. Among them, those highlighted in yellow show enriched expression in male germ cells relative to other cell types in the human body. The asterisk indicates a gene deletion or mutation that leads to defects in spermatogenesis and male infertility.

Thus, differential regulation of the expression of PP2A subunit isoforms may influence the composition of PP2A heterotrimers in maturing male germ cells and thus allow the formation of the PP2A heterotrimers specifically involved in the main stages of spermatogenesis: mitosis, meiosis, and spermiogenesis. An overview of the PP2A subunits expressed in the course of spermatogenesis and their potential participation in the formation of PP2A heterotrimers is presented in Fig. 4. The expression profile of PP2A during spermatogenesis may help to get a better understanding of the role of PP2A in male germ cell production.

## PP2A function during sperm maturation – importance for male fertility

Interestingly, most of the PP2A subunit isoforms are detected in the proteome of human ejaculated spermatozoa (75), in line with a potential role of PP2A in the acquisition of sperm fertilization potential, which is well established to be finely tuned by phosphorylation/dephosphorylation events. This chapter will provide an update regarding studies that indicate a role of PP2A in the process of sperm functional maturation.

The spermatozoon is structurally formed when it leaves the testis but only becomes motile after functional activation processes which occur during its transit through the male and female reproductive tracts. These processes involve a complex network of signaling pathways triggered by ion fluxes and ultimately leading to the hyperphosphorylation of sperm proteins through a finely regulated balance between kinase and phosphatase activities mobilized by the sperm. The involvement of serine/threonine phosphatases in sperm functional activation has been recently detailed and summarized by Ferreira and colleagues (13). Most studies that have so far examined the role of sperm phosphatases are based on pharmacological inhibitors used at concentrations that inhibit both PP2A and PP1 (for review see (13)). These phosphatase inhibitors are OA, calyculin A (CA), and endothall (E). OA and CA were initially isolated from marine sponges such as *Halichondria okadai* and *Discodermia calyx*, respectively, while E is a synthetic herbicide. Inhibition of PP2A and PP1 activities using these compounds increases protein phosphorylation and motility of spermatozoa from several mammalian species (mouse, bovine, boar, human, and monkey) (for review see (13)). The demonstration of the involvement of PP2A in sperm maturation comes from a few studies where PP2A was selectively inhibited by low nanomolar concentrations of OA and E (OA: PP2A, IC_50_ = 0.1 nM, PP1, IC_50_ = 15–20 nM; E: PP2A, IC_50_ = 90 nM, PP1, IC_50_ = 5 μM). Selective inhibition of PP2A in bovine and hamster spermatozoa with 0.1 and 5 nM of OA, respectively, increases the percentage of motile and hypermotile spermatozoa (76, 77). In addition to its role in sperm motility, PP2A was also shown to trigger the acrosomal reaction, which corresponds to the fusion of the outer acrosomal and plasma membranes of the spermatozoa and constitutes a prerequisite for unmasking a set of sperm membrane proteins, which are engaged in oocyte recognition and interaction. Hence, inhibition of PP2A by endothall (90 nM) was shown to induce a rapid increase in the percentage of acrosome-reacted human spermatozoa (78). In consistency with the above results, the endogenous activity of PP2A was shown to decrease markedly both in bovine and human spermatozoa during their transit and maturation throughout the male and female genital tracts, such downregulation of PP2A activity being concomitant with the well-established increase of sperm protein phosphorylation (77, 78). Some cues to define the mechanisms underlying the physiological inhibition of PP2A in spermatozoa were provided by studies indicating the role of the Src family kinases (79, 80) and post-translational modifications of the catalytic subunit of PP2A (methylation (L309) and dephosphorylation (Y307); (77)). Collectively, these studies delineate the critical involvement of PP2A in the process of sperm maturation and establish a framework for future investigations to better define the molecular mechanisms supporting PP2A regulatory pathways within sperm.

## Concluding remarks

Although the role of PP2A in male germ cells has long been underestimated, recent studies have now demonstrated its involvement in proper spermatogenesis and sperm post-testicular functional activation. These studies suggest that PP2A is involved not only in the stages of mitosis and meiosis but also during spermiogenesis to support the differentiation of spermatids into spermatozoa. Given such critical functions, the deregulation of PP2A in male germ cells merits investigation in human cases of spermatogenesis defects and male infertility.

Expression of the various isoforms of the regulatory subunits of PP2A and their inhibitors is specifically and dynamically regulated throughout spermatogenesis for each isoform. Based on current knowledge, spermatogenesis is the first physiological system for which such variation in PP2A expression has been observed. This amazing panoply of subunit isoforms coupled with their physiological regulation can lead to the formation of functional PP2A heterotrimers of distinct composition in spermatogonia, spermatocytes, and spermatids, with specific roles in the regulation of mitosis, meiosis, and spermiogenesis together with post-testicular sperm maturation/activation. The current development of a growing number of molecules that can specifically target PP2A-B55 and PP2A-B56 heterotrimers and result in their inhibition or stimulation will undoubtedly contribute to a better understanding of their respective roles in male germ cells. In the future, such compounds could potentially be used to restore spermatogenesis and sperm maturation in infertile patients by specifically targeting one or more PP2A heterotrimers. Conversely, this gain of knowledge will also fuel the development of alternative contraceptive strategies, which, to date, constitute an important societal demand.

## Declaration of interest

The author declares that there is no conflict of interest that could be perceived as prejudicing the impartiality of the work reported.

## Funding

This work was supported by the Institut National de la Santé et de la Recherche Médicale (INSERM), the Centre National de la Recherche Scientifique (CNRS), and the Université Grenoble-Alpes (UGA).
